# Inkjet-printed stretchable and low voltage synaptic transistor array

**DOI:** 10.1038/s41467-019-10569-3

**Published:** 2019-06-18

**Authors:** F. Molina-Lopez, T. Z. Gao, U. Kraft, C. Zhu, T. Öhlund, R. Pfattner, V. R. Feig, Y. Kim, S. Wang, Y. Yun, Z. Bao

**Affiliations:** 1 Department of Chemical Engineering, Stanford University, 443 Via Ortega, Stanford, CA 94305-4125 USA; 2 Department of Materials Science and Engineering, Stanford University, 496 Lomita Mall, Stanford, CA 94305-4034 USA; 3 Department of Electrical Engineering, Stanford University, 350 Serra Mall, Stanford, CA 94305 USA; 4 Department of Natural Sciences, Mid Sweden University, Holmgatan 10, Sundsvall, 852 30 Sweden; 5 Samsung Advanced Institute of Technology, 130 Samseong-ro, Suwon, 16678 South Korea; 6 Present Address: Department of Materials Engineering, KU Leuven, Kasteelpark Arenberg 44, 3001 Leuven, Belgium; 7 Present Address: Cavendish Laboratory, University of Cambridge, JJ Thomson Avenue, Cambridge, CB3 0HE UK; 8 Present Address: Institute of Materials Science of Barcelona (ICMAB-CISC), Campus de la UAB, 08193 Bellaterra, Spain; 9 Present Address: Institute for Molecular Engineering, University of Chicago, 5640S Ellis Avenue, Chicago, IL 60637 USA

**Keywords:** Chemical engineering, Electronic devices, Polymers

## Abstract

Wearable and skin electronics benefit from mechanically soft and stretchable materials to conform to curved and dynamic surfaces, thereby enabling seamless integration with the human body. However, such materials are challenging to process using traditional microelectronics techniques. Here, stretchable transistor arrays are patterned exclusively from solution by inkjet printing of polymers and carbon nanotubes. The additive, non-contact and maskless nature of inkjet printing provides a simple, inexpensive and scalable route for stacking and patterning these chemically-sensitive materials over large areas. The transistors, which are stable at ambient conditions, display mobilities as high as 30 cm^2^ V^−1^ s^−1^ and currents per channel width of 0.2 mA cm^−1^ at operation voltages as low as 1 V, owing to the ionic character of their printed gate dielectric. Furthermore, these transistors with double-layer capacitive dielectric can mimic the synaptic behavior of neurons, making them interesting for conformal brain-machine interfaces and other wearable bioelectronics.

## Abstract (web summary)

The development of novel low-cost fabrication schemes for realizing stretchable transistor arrays with applicability in wearable electronics remains a challenge. Here, the authors report skin-like electronics with stretchable active materials and devices processed exclusively from ink-jet printing.

## Introduction

Skin-like bioelectronics enables user-imperceptible integration of electronics directly on the body for applications, such as sensing biological signals or enhancing biological functions^1^. Hence, it requires the development of devices with the same form factor, mechanical properties, and chemical characteristics of human skin^2^. This includes large area, softness, stretchability, and biocompatibility. The two main strategies to achieve stretchable electronics are patterning inorganic materials on pre-strained substrates to form in-plane and out-of-plane serpentines or buckles^3,4^; and the use of intrinsically stretchable materials achieved by molecular engineering, nano-structuring, or the use of composites^5^. Although the former approach often yields higher performance devices owing to the incorporation of inorganic crystalline materials, it entails significant patterning complexity. The latter approach, on the other hand, requires far less complex patterning, and thus possesses superior potential for device interconnection and integration with high device density^6^.

Intrinsically stretchable materials are usually based on organic materials that can be processed from solution^5^. A particularly promising branch of solution-based processing techniques is printing, which enables scalable additive patterning over large areas, and at low fabrication cost per surface area^7^. By combining printing techniques with liquid inks of functional organic materials, direct additive fabrication of stretchable electronics with these characteristics can be realized.

Although the traits of printing are desirable for skin electronics, printing techniques have seldom been explored for the integration of intrinsically stretchable materials. The lack of overlap between these two fields thus far is due to the challenges associated with the formulation of such new materials into inks able to be patterned and stacked in a reliable way. Current fabrication procedures for skin-like electronics therefore still strongly rely on techniques such as spin coating, photolithography, shadow mask, or evaporation of active materials that restrict devices to small areas, as well as transfer steps that involve complicated alignments^6,8–13^.

Among the most commonly used printing techniques, inkjet (IJ) printing stands out due to its good resolution (~μm); tight control of deposited volume; digital, maskless, and additive character (that enables material usage minimization and process flow simplicity); and non-contact nature. Moreover, the simplicity and low cost of commercially available IJ printers for electronics has vastly popularized its usage^14–16^. However, IJ printers typically use small nozzle size to enable high resolution as well as controlled deposition, with the potential drawback of nozzle clogging. Reliable IJ printing therefore requires careful design of ink formulations. Despite its limitations, many groups have achieved IJ-printed field effect transistors (FETs) on both rigid and flexible (non-stretchable) substrates using inks of organic semiconductors^16,17^, as well as semiconducting single-walled carbon nanotubes (SC-SWCNTs)^18–20^. Although SC-SWCNTs must be carefully separated from their metallic counterparts and also tend to aggregate strongly in solution, they present greater thermal stability and higher mobility than organic semiconductors (as high as 30 cm^2^ V^−1^ s^−1^ with on-current to off-current ratio, *I*_on_/*I*_off_ > 10^4 ^^12,21,22^). Moreover, SWCNTs form networks that have been proven to maintain electrical contact under mechanical strain, making them an excellent semiconductor material for skin electronics^11–13,23^.

In most of the reported examples of SWCNTs FETs, however, only one or few functional layers of the final device were patterned by printing, with the deposition/patterning of the gate dielectric often being the limiting factor. Notably, fully-printed SC-SWCNTs FETs, which also include printed/patterned gate dielectrics, comprised of BaTiO_3_−polymer composites^20,24,25^, poly(vinyl phenol)/poly(methyl silsesquioxane) (PVP/pMSSQ) blends^26^, ionic gels^27^, or polyfluorinated electrolytes^28^ have been only recently reported. Polyfluorinated electrolyte can form thermally stable and mechanically flexible gate dielectrics^28^. This type of polymers as well as ion gels exhibit the electric double layer (EDL) effect, which results in high gate capacitance even for thick dielectric layers. This high capacitance facilitated low-voltage FET operation of few volts^9,10,13,21,27,28^. However, ion gel electrolytes have poor mechanical strength and are sensitive to moisture. The mentioned demonstrations utilized aerosol-jet printing or a combination of roll-to-roll gravure and IJ printing to fabricate devices on glass and plastic substrates. A fabrication scheme exclusively utilizing IJ printing for all the device functional layers would lower processing complexity and further enhance device integration and interconnection.

Here, we report on the additive fabrication of skin-like electronics by simultaneous deposition, patterning, and aligning of stacks of intrinsically stretchable active materials exclusively using IJ printing. Arrays of FETs are prepared by stacking the following materials: stretchable conductive poly(3,4-ethylenedioxythiophene)-poly(styrenesulfonate) (PEDOT:PSS)^29^ as the source/drain/gate electrodes and interconnects; a network of SC-SWCNTs as the semiconductive channel; and a fluorinated polymer consisting of a solid-state ionic poly(vinylidene fluoride-*co*-hexafluoropropylene) (PVDF-HFP) as the gate dielectric. Our PVDF-HFP offers the low-voltage operation advantages of EDLs^28,30,31^ without the poor mechanical, chemical, and thermal stability of ion gel counterparts. Ultra-low-voltage operation is desirable for wearables that need to be powered by small batteries or energy harvesters, as well as for devices in direct contact with human body. The final devices present excellent electrical performance in ambient conditions, with average mobility of 27 ± 5 cm^2^ V^−1^ s^−1^, *I*_on_/*I*_off_ > 10^4^ and maximum transconductance of 47 ± 9 μS. Those parameters are superior to the state of the art for flexible/stretchable CNT-based FETs. Moreover, the electrical nature of the SC-SWCNT network and PVDF-HFP gate dielectric make the FETs strain independent because the tube-to-tube charge transport in SWCNT networks is strain and channel geometry independent (after initial cycling^11,23^), and the EDL-based capacitance per surface area of the PVDF-HFP is thickness independent above the thickness of the EDL (ca. 100 nm)^30,31^. Indeed, the reported devices present mechanical stretchability, with electrical performance insensitive to strains up to 20% both along the direction parallel and perpendicular to the FET channel. These properties make the fabricated circuits suitable for skin-like applications. In particular, we show that each transistor emulates the synaptic behavior of neurons^32,33^, which situates this system as an excellent candidate for future in vivo neural interfaces. Due to its outstanding mechanical and electrical properties, the system reported here pushes forward the additive and scalable fabrication of large-area, skin-like electronics for bioelectronics applications.

## Results

### Fabrication process flow

**Fig. 1:**
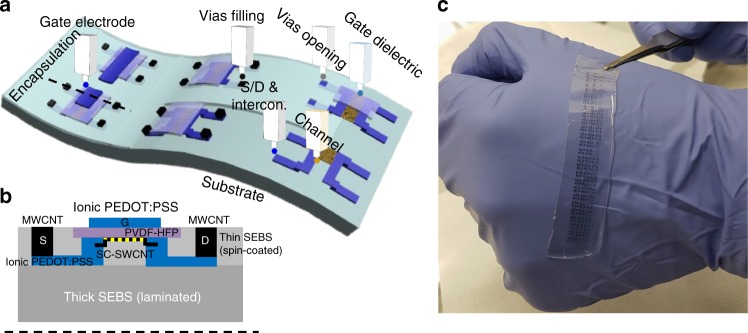
General concept and design. **a** Sketch of the intrinsically-stretchable array of transistors representing how each active material of the transistor was additively fabricated with the same inkjet (IJ) printing method. These materials were used to form: source (S), drain (D), and gate (G) electrodes, source and drain interconnections, gate dielectric, channel, and through encapsulation vias. The dashed line corresponds to the location of the cross-section shown in **b**. **b** Sketch of the cross-section of a device, where the each layer material is labeled. **c** Picture of an array of transistors IJ printed over a large area and bent over a hand

**Fig. 2:**
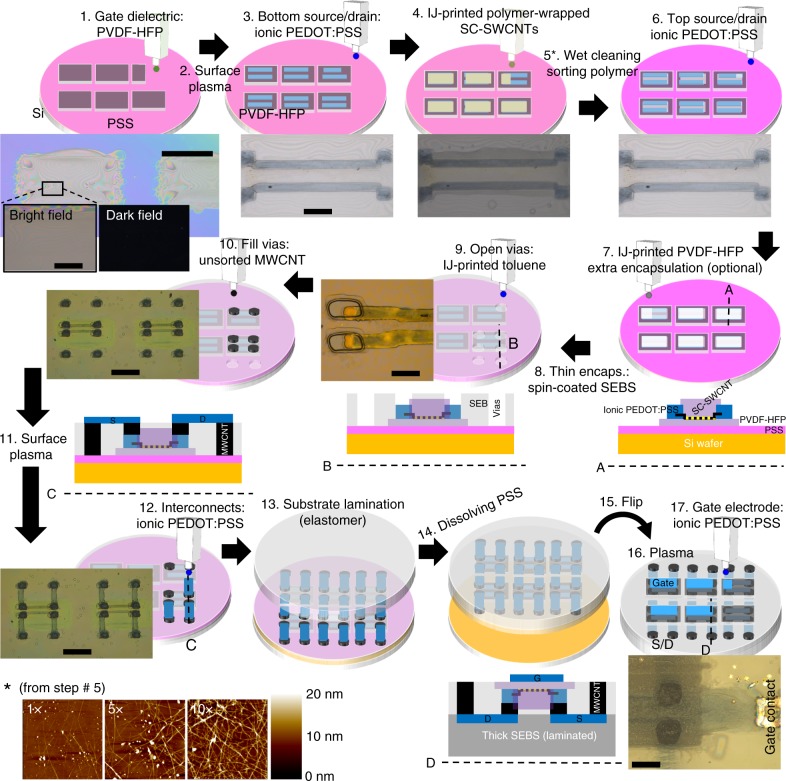
Detailed fabrication of the inkjet (IJ)-printed stretchable single-walled carbon nanotube-field effect transistor (SWCNT-FET) arrays. The process steps are numbered and presented in chronological order of fabrication along with a simplified three-dimensional (3D) or cross-section view sketch. Representative optical pictures of the devices after some steps are also added for clarity. The atomic force microscopy (AFM) amplitude topographies in the inset at the bottom-left corner of the figure corresponds to step #5. It shows how the SWCNT density (number of tubes per surface area) of IJ-printed semiconductor (SC)-SWCNTs networks can be easily tuned by the number of printing passes: the network resulting from 1, 5, and 10 printing passes on a SiO_2_ substrate is shown after removal of the sorting polymer. Scale bar = 200 μm in steps: 1 (zoom), 3, 9, and 17. Scale bar = 1 mm in steps: 1, 10, and 12

Arrays of soft and stretchable FETs like the one shown in Fig. 1 were fabricated by layer-to-layer additive deposition and direct patterning of various intrinsically stretchable materials using IJ printing. The process flow (Fig. 2) was specially designed to ensure chemical compatibility between the different layers by using orthogonal solvents and localized deposition. We also target the minimization of thermal stresses by limiting the maximum process temperature to 160 °C first (to anneal the gate dielectric), and to 70 °C later on when more layers were added to the system. The processing flexibility of IJ printing was paramount to enable this process. Owing to the digital and maskless character of IJ printing, the number, layout, and arrangement of the fabricated devices were easily customized by modifying the digital file input to the printer. As a proof of concept of our system, we designed transistors with channel width and length of 1 mm and 50–100 µm, respectively, arranged in arrays of different size ranging from 2 × 3 to 3 × 20 devices with a device density of 20 devices cm^−2^. The surface area of a single transistor (without interconnections) was of 2 mm^2^, corresponding to the size of dielectric island. Although these devices are relatively large and widely spaced compared to the maximum integration achievable by IJ printing, which is capable of material placement accuracy of ±5 μm, their dimensions are still smaller than most organic and CNT-FETs or arrays of FETs previously reported^8,17,20,24–28^.

To facilitate the handling of the system throughout the different fabrication steps, a Si wafer was employed as a rigid holder. The wafer was coated with a water-soluble layer of poly(sodium 4-styrenesulfonate) (PSS) used later on as a sacrificial layer for release of the stretchable system from the rigid substrate. Next, a thick ionic gate dielectric (PVDF-HFP) was IJ printed as square islands (step #1). The thickness-independency of our gate dielectric (Supplementary Fig. 1a) enables the elimination of gate current leakage by printing thick gate dielectric films without decreasing the FET channel capacitance value. Due to the typical unevenness of printed layers, the gate dielectric needed to be thicker than 1.75 μm to avoid gate current leakage (Supplementary Fig. 1b). Optimization of the printing parameters was crucial to ensure a smooth dielectric surface (see Methods section and Supplementary Fig. 1c). Silver contact pads were optionally IJ printed onto the PSS layer on positions adjacent to the dielectric islands to facilitate the final external probing of the devices (this step is not shown in the figure, but a silver contact pad connected to the gate electrode can be seen in the picture of step #17). After annealing the gate dielectric (and optionally the silver contact pads) at 160 °C for crosslinking, its surface was activated with oxygen plasma (step #2) to improve the wettability of the ionic PEDOT:PSS that was immediately IJ printed on it to define the source and drain electrodes (in a bottom contact configuration) in step #3.

Next, a toluene-based ink composed of SC-SWCNTs and conjugated polymer used for sorting was IJ printed on the channel and the area extending over source and drain during step #4^34^. This ink was specially formulated for IJ printing as described in the Methods section. The sorting polymer was removed by immersion of the devices in a mixture of toluene and trifluoroacetic acid (TFA) in step #5. IJ printing allows for controlled deposition and simultaneous patterning of the SWCNTs network. The inset of Fig. 2 shows how the tube density (number of tubes per surface area) of an IJ-printed SWCNTs network can be increased with the number of printing passes. Denser networks produced higher currents up to a limit at which the substrate was saturated with SWCNTs. Beyond this limit (corresponding to the 10× layer shown the picture), no improvement in current was observed (Supplementary Fig. 2), likely because the extra printed tubes were removed during the sorting polymer removal process.

The layer-to-layer alignment capabilities of IJ printing allowed for printing of the source and drain electrodes on top of the previously-printed electrodes (step #6), sandwiching the SC-SWCNTs in between. This top and bottom double contact configuration is beneficial for reducing contact resistance as it has been demonstrated before^35^.

Following, in step #7 the channel was partially encapsulated by locally printing PVDF-HFP dielectric on top and annealing at 70 °C. This step improved the FETs performance and was enabled by the non-contact and additive character of IJ. Interestingly, other authors have also reported advantages in electrical performance for CNT-FETs when using fluorinated polymers to cover the CNTs^36^. A system-level encapsulation was then produced by spin coating a film of thermoplastic styrene ethylene butylene styrene (SEBS) elastomer from toluene (step #8). We provided the system with the potential to accommodate more complex circuitry in future applications by enabling a second “metallization” layer on the SEBS encapsulation. This layer allows easy interconnection between different transistors in the array^37^. IJ printing of pure toluene was then employed to open via holes on the SEBS encapsulation above the source and drain electrodes to access them (step #9). An extra set of vias were also open away from the source and drain, beyond the area occupied by the gate dielectric, to facilitate the final connection of the devices through the backside. The vias were filled with a dimethylformamide (DMF)-based conductive ink containing graphene and unsorted multiwall CNTs (MWCNT) to provide electrical conductivity, and polyacrylonitrile (PAN) polymer to prevent aggregation^38^ (step #10). This material was used to fill the vias instead of PEDOT:PSS due to the hydrophobicity of PAN, which protected the channel from water absorption in subsequent steps involving water immersion. Moreover, the geometry of the vias (roughly 200 μm × 200 μm in cross-section and only ~1 μm in length) leads to low resistance (<kΩ) even for this low conductivity material that contains high concentration of dielectric PAN.

After filling the vias, the encapsulation layer of SEBS was shortly treated with oxygen plasma (step #11) to facilitate the printability of the stretchable ionic PEDOT:PSS traces that interconnect source and drain electrodes with the external vias (step #12).

A stretchable substrate (SEBS of 1.5 mm in thickness or Tegaderm^TM^ from 3M of 20 μm in thickness) was then laminated in step #13, followed by soaking in water to dissolve the water-soluble PSS sacrificial layer and release the devices from the rigid Si substrate onto the stretchable substrate (step #14).

Finally, the substrate was flipped (step #15) and subjected to a short oxygen plasma treatment (step #16) before the gate electrode was IJ printed from ionic PEDOT:PSS (step #17). For improved reliability, the gate dielectric can be optionally made thicker in order to prevent leakage by repeatedly printing PVDF-HFP prior to the deposition of the gate electrode (not shown in Fig. 2). Since the gate low-frequency (<10 Hz) capacitance is independent of thickness, making a thicker layer does not impact it. It is worth noting that IJ printing helps saving extra alignment steps when different materials and layouts are sequentially printed without the need of moving the devices from the printer stage. This is the case for printing the CNTs channel in our system right after defining the PEDOT:PSS source and drain electrodes, or for filling via holes immediately after opening them. Those processes would have required extra alignment if they were carried out with a different fabrication technique. Most importantly, the dimensional stability of soft materials is typically poor. As a result, after thermal annealing, lamination, or releasing steps, significant and unpredictable expansion/shrinkage of the layout may occur, making subsequent accurate mask alignment difficult. This dimensional mismatch becomes more critical for larger circuits or arrays. Contrary to masking techniques, IJ printing allows for quick re-measurement of the features of the system and automatic adjustment of the layout to accommodate for dimensional changes.

### Electrical characterization

**Fig. 3:**
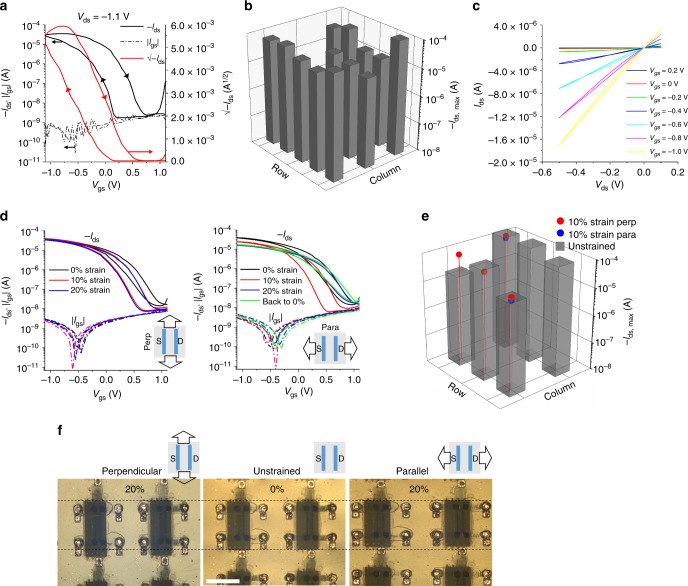
Electrical characterization of the inkjet (IJ)-printed stretchable field effect transistors (FETs). **a** Transfer curve and square root of source–drain current √−*I*_ds_ vs. gate voltage (*V*_gs_) of a representative IJ-printed intrinsically stretchable single-walled carbon nanotube-FET (SWCNT-FET). **b** Map of the max drain–source current (−*I*_ds,max_) in saturation regime for each FET in an array of 5 × 3. The non-plotted devices were not functional. **c** Output characteristics at different gate voltages. The *W*/*L* of the all the devices was 1000 µm/50 µm. **d** Overlapped transfer curves of the IJ-printed SWCNT-FET subjected to different strain conditions along the two main directions: perpendicular (left) and parallel (right) to the channel length. **e** Map of −*I*_ds,max_ in saturation regime for each FET in an array of 3 × 2 unstrained and stretched 10% in the directions perpendicular (perp) and parallel (para) to channel length. Current measurements under strain are available only for some representative devices due to setup limitations and yield loss resulting from the heavy handling involved in the stretching experiment. **f** Optical microscope images of some transistors stretched at 20% strain along the two main directions. The dashed lines serve to highlight the change in size of the stretched transistors when compared to the reference transistor (0% strain) in the middle. The scale bar is 1 mm

The fabricated devices displayed excellent transistor behavior in ambient conditions (Fig. 3a), and remain functional after dwelling in ambient conditions for few days (Supplementary Fig. 3). The ratio between on-current and off-current was >10^4^ for very low gate and drain voltage of 1 and 1.1 V, respectively, and the gate leakage current was negligible.

The mobility *µ* can be calculated from the FET saturation regime using the curve (*I*_ds_)^1/2^ vs. *V*_gs_ in Fig. 3a as follows:

1 $$\mu = \frac{{2L}}{W}\frac{1}{C}\left( {\frac{{\partial \sqrt {I_{{\mathrm{ds}}}} }}{{\partial V_{{\mathrm{gs}}}}}} \right)^2,$$

where *W* and *L* are the channel width and length (1000 and 50 μm, respectively), and *C* is the capacitance per surface area of the gate dielectric. In our devices, this capacitance corresponds to the EDL of the IJ-printed ionic PVDF-HFP. Its value, 250 ± 30 nF cm^−2^, was measured at quasi-direct current (DC) conditions. This value is similar to previously reported values for spin-coated films of the same material^30,31^. It is worth noting that the mobility value for CNT-FETs is usually underestimated when using a capacitance value calculated or measured with standard parallel-plates. This is due to the limited surface coverage and cylindrical geometry of random SWCNTs networks, which differ from a continuous electrode assumed in the parallel-plate model. On the other hand, although more rigorous capacitance models have been reported to calculate the intrinsic capacitance of SWCNT-based devices^19,22,39^, they cannot be used in this case because they have been designed for standard (non-ionic) dielectrics.

The high clockwise hysteresis suggests that the slow-moving ions in the dielectric layer do not have time to reach equilibrium when sweeping *V*_gs_, which introduces uncertainty in the extracted value of mobility in the saturation regime. Indeed, the extracted average mobility from the forward curve was 24 ± 5 cm^2^ V^−1^ s^−1^ (for *V*_ds_ = −1.1 V), significantly different from the backward curve mobility, 12.5 ± 2.1 cm^2^ V^−1^ s^−1^ (see Methods section for details and Supplementary Fig. 4a). However, the curve (*I*_ds_)^1/2^ vs. *V*_gs_ is linear only for the forward voltage sweep, which makes this part of the curve more reliable for extracting the FETs parameter. Most devices in the fabricated arrays presented similar characteristics with comparable maximum on-current (within one order of magnitude, Fig. 3b). The existing on-current variations result from the high device variability introduced by the usage of non-commercial materials, a novel non-standard processes and differences in contact resistance arising from an imperfect probing system.

As shown in the FET output characteristics displayed in Fig. 3c, the hysteresis is minimized when the gate voltage *V*_gs_ is held at a fixed value for a prolonged period of time of tens of seconds and *V*_ds_ is swept instead. The mobility can also be extracted in the FET linear regime from the output curve corresponding to *V*_gs_ = −1 V as:

2 $$\mu = \frac{L}{W}\frac{1}{C}\frac{1}{{\left| {V_{{\mathrm{gs}}} - V_{{\mathrm{th}}}} \right|}}\frac{{\partial I_{{\mathrm{ds}}}}}{{\partial V_{{\mathrm{ds}}}}},$$

where the threshold voltage *V*_th_ was estimated to be −0.2 V (see Methods). Similar mobility values of 27 ± 5 cm^2^ V^−1^ s^−1^ and 28 ± 5 cm^2^ V^−1^ s^−1^ were calculated in the linear regime for both forward and backward sweeping of the *V*_ds_ voltage, respectively (Supplementary Fig. 4b). These values match the forward mobility in the saturation regime, adding more confidence to it. The extracted mobility is high enough for most low-speed applications foreseen for this kind of devices in electronic skin.

In addition to the high measured mobility, another parameter supporting the promising potential of this type of devices for low-voltage applications is their high transconductance, which is defined as:

3 $$g_{\mathrm{m}} = \left. {\frac{{\partial I_{{\mathrm{ds}}}}}{{\partial V_{{\mathrm{gs}}}}}} \right|_{\left| {V_{{\mathrm{gs}}}} \right| > \left| {V_{{\mathrm{th}}}} \right|}.$$

The maximum transconductance extracted from the transfer curve for the whole range of |*V*_gs_| > |*V*_th_| (Supplementary Fig. 4c) was comparable for both forward and backward sweeping, displaying an average value of 47 ± 9 and 41 ± 7 μS, respectively, for a *V*_ds_ as low as −1.1 V and a channel *W*/*L* = 1000 μm/50 μm.

Because the devices were manufactured with intrinsically stretchable materials, their electrical properties did not significantly degrade when the array was subjected to mechanical strain. As depicted in Fig. 3d–f, the drain–source current *I*_ds_ of the transistors was maintained when stretched up to 20% strain in the directions perpendicular to the current flow across the channel. On the other hand, some current degradation was observed when the stretching was performed parallel to the current flow direction. This degradation was mostly due to irreversible contact damage, as supported by the fact that the current remained unchanged after relaxing back the device to 0% strain (referred as “back to 0%” in the curve). Regarding the threshold voltage, the variations observed between different measurements are attributed to the memory effect introduced by the ionic gate dielectric, rather than to the strain. This memory effect makes the threshold voltage dependent on the time and voltage history of the applied voltages (Supplementary Fig. 5). Beyond 20% strain, the devices failed mainly due to cracking of the gate electrode (Supplementary Fig. 6) and interconnections. Although many of the envisaged skin-electronics applications require devices to operate at strains no larger than the 20–30% achievable by human skin^40^, alternative gate materials and encapsulations are currently being researched in order to expand the stretching margin of our devices for other alternative applications such as robotic skin.

### Application as synaptic transistors

**Fig. 4:**
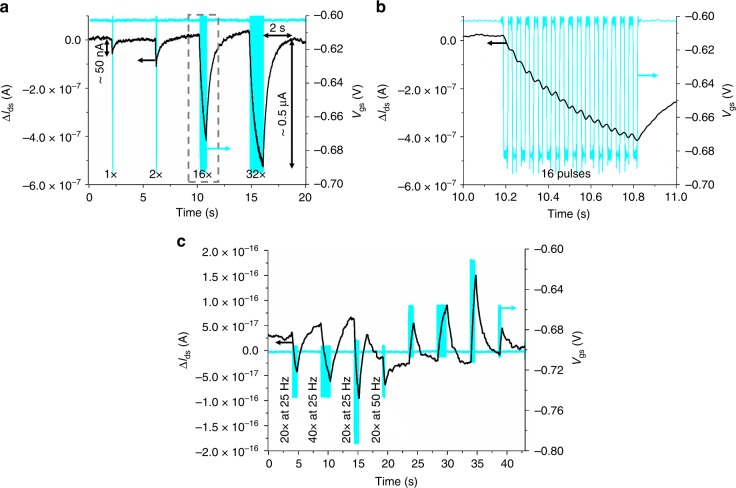
Synaptic behavior of the inkjet (IJ)-printed stretchable field effect transistors (FETs). **a** Source–drain current variation (Δ*I*_ds_, on the left axis) response over time to small gate voltage pulses (*V*_gs_, on the right axis) that imitates neuron presynaptic potential spikes for FETs with *W*/*L* = 1000 µm/50 µm. The pulses consist of a square signal of −80 mV of amplitude (on top of an initial biasing voltage of −0.6 V applied for long enough time to ensure the full formation of the channel), 25 Hz of frequency, and duty cycle of 50%. The drain–source voltage was held at −1.1 V. The source–drain current response is tested for consecutive trains of 1, 2, 16 (magnified in **b**) and 32 gate voltage pulses, displaying typical postsynaptic current variation that increases with the number of pulses and relaxes in their absence (the higher the number of pulses, the longer the relaxation time). **b** Zoom-in on the part of the signal delimited by the dashed box in **a**. The ripple in Δ*I*_ds_ observed for each individual pulse of only −80 mV highlights the good voltage resolution of the devices. **c** Source–drain current variation (Δ*I*_ds_, on the left axis) over time for the same devices when voltage pulse trains are applied to the gate. The pulse trains have a duty cycle of 50% and are inputted in such a way that each train differs from the first one (control) in either: (1) number of pulses (40 pulses for the 2nd train vs. 20 for the control), (2) amplitude (−80 mV for 3rd train vs. −40 mV for control), or (3) frequency (50 Hz for the 4th train vs. 25 HZ for control). The source–drain current responds to all three factors in agreement with short-term synaptic plasticity, that is, the higher the number of pulses, their amplitude and their period, the higher the produced current variation and the longer the time needed for the devices to relax back to equilibrium. A second set of pulse trains with opposite voltage sign generates also an opposite signed current change, demonstrating that the devices are capable of both symmetrical excitatory and inhibitory postsynaptic current

Owing to its mechanical softness of ~7 MPa (Supplementary Fig. 7), stretchability, and operation in ambient conditions, the reported system presents high potential for interfacing with biological systems. Furthermore, compared to other skin-electronic devices, this system can be operated at low voltage and possesses high transconductance as a result of the ionic nature of the gate dielectric. Those characteristics enable these devices to mimic synaptic neuron behavior with the potential for future in vivo interfacing with neurons as artificial synapse. Figure 4a confirms the synaptic behavior of the reported transistors. The drain–source current, *I*_ds_, reversibly responds to pulse trains of very small voltage input at the gate (Fig. 4b), reproducing typical excitatory postsynaptic current (EPSC) found in biological dendrites. The low voltage (of tens of mV) applied at the gate emulates the presynaptic action potential spikes fired by a biological axon and a presynaptic membrane. This finding suggests that the migration of the ions in the gate dielectric is comparable to the dynamics of neurotransmitters in the synaptic cleft, which allows chemical transmission but is electrically insulating^32^. The nature of the ionic gate dielectric underlies the mechanism of short-term plasticity (STP) as previously reported in other synaptic devices using ionic gel^32^. Synaptic plasticity is defined as the variation in the strength of a synapse’s response over time upon external stimuli. STP can modify a synapse for a time scale from tens of milliseconds to a few minutes and it plays a role in encoding temporal information in auditory and visual signals^41^. In our transistors, as a result of a single gate voltage spike (−80 mV during 20 ms), ions migrate to the dielectric interface, but quickly relax back to their initial equilibrium state when the voltage pulse ends. This produces a channel current that gradually changes in ~ 50 nA and immediately comes back to its initial state in roughly 300 ms. It is known that short-term synaptic enhancement can be attained when several spikes arrive in rapid succession^32,33^. Upon application of 32 quick consecutive negative voltage pulses (−80 mV) at 25 Hz at the gate of the transistors, the channel current amplitude changes in 500 nA due to the accumulation of ions at the interfaces of the dielectric. Such large concentration of ions at the dielectric interface requires longer time to relax back to equilibrium, and lead to a short-term memory-like channel current that extend ~2 s beyond the application of the pulses. We demonstrated that in agreement with STP, the greater the number of pulses (Fig. 4a), and their period and amplitude (Fig. 4c), the greater the channel current value changes and the time needed to relax back to equilibrium. Finally, besides EPSC, these devices are equally capable of symmetrical inhibitory postsynaptic current (IPSC), where positive voltage spikes make the current to shift on the opposite direction than negative voltage spikes (Fig. 4c).

## Discussion

Stretchable FET arrays and interconnections have been fabricated by IJ printing using intrinsically stretchable electronic materials, namely ionic PEDOT:PSS, ionic PVDF-HFP, and SC-SWCNTs networks. Each functional material was formulated as an ink and deposited/patterned at ambient conditions without the need of any mask.

The devices exhibit good transistor performance in ambient conditions with an average mobility of 27 ± 5 cm^2^ V^−1^ s^−1^, *I*_on_/*I*_off_ > 10^4^ and maximum transconductance of 47 ± 9 μS. The FETs were capable of sub-volt operation owing to the double-layer behavior of its ionic PVDF-HFP gate dielectric. Such behavior provided high capacitance values and low leakage without the challenges associated with fabricating pinhole-free, thin and uniform printed dielectric layers. Owing to the intrinsically stretchable nature of the materials used, the array survived in-plane stretching to up to 20% strain. However, the main components of the FETs should be capable of surviving even greater strains (see refs. ^11,29^ and Supplementary Fig. 8). Unfortunately, the potential of the system to stretch beyond this limit could not be evaluated for the current architecture due to decreasing yield beyond 10% strain, as a result of interconnects and gate electrode cracking (Supplementary Fig. 6). The cracking of the interconnections was especially severe in the direction parallel to the channel length, which was also perpendicular to the dominant printing direction. Further improvements in printing technology (e.g., by allowing equivalent printing in both *x* and *y* directions) can reduce the anisotropy of printed layers and the risk of cracking in the direction perpendicular to printing. Likewise, improving the film thickness uniformity and edge straightness of the gate electrode (Supplementary Fig. 6), and/or encapsulating it could also contribute to enhancing the stretchability of the system.

This system is applicable for various scenarios in wearables, skin electronics, or bioelectronics where low operating voltage, high mobility, air/water stability of electrical characteristics, softness, conformability, or stretchability are required. The main limiting factor for the system is its switching speed, which is impeded by the slow motion of the ions in the polymeric gate dielectric. However, such slow ionic-like response is desirable for other applications such as synaptic transistors, which intend to mimic neuron-to-neuron communication. Indeed, we demonstrated that the ionic nature of the reported gate dielectric accurately emulates STP, supporting both excitatory and inhibitory postsynaptic currents, for very small input voltages of 40 mV. Therefore, this class of devices can potentially be used in direct contact with neurons (especially when considering that PEDOT:PSS has already been shown to have very low contact resistance with neurons^42^), enabling new advances in neuromorphic engineering and brain–machine interfaces.

## Methods

### Device fabrication

The IJ printer used was a Dimatix Material Printer DMP-2831 from Fujifilm with 10 pL droplet size cartridges (standard and chemically resistant liquid crystal polymer (LCP) cartridges).

*Rigid substrate holder preparation*: A solution of PSS (*M*_w_ ~200 kDa, 30% wt. in H_2_O from Aldrich, CAS: 25704-18-1) was diluted (1:3)_V_ in deionized water, degassed for 5 min, and filtered through a 0.45 µm pore-size nylon filter before spin coating it at ambient conditions at 2000 rpm for 30 s (acceleration 1000 rpm s^−1^) on a doped Si wafer previously treated with plasma oxygen (Technics Micro-RIE series 800) for 1 min at 150 W. The layer was annealed for 30 min at 150 °C under vacuum (central vacuum line), cooled down slowly, and stored in vacuum.

*Gate dielectric*: A solution of the elastomer PVDF-HFP (3M™ Dyneon™ FC2176 Fluoroelastomer FE) in *N*-methyl-2-pyrrolidone anhydrous (Sigma-Aldrich, CAS:872-50-4) at a concentration of 10 mg ml^−1^ was prepared by mixing the components and stirring at 80 °C for 30 min. The solution was used to fill an LCP cartridge (to minimize solvent–cartridge interaction) after passing through a 0.2 µm pore-size nylon filter (Millipore Millex-GN 13 mm in diameter) or a 0.4 µm pore-size PTFE filter. We observed significant differences on the final layer quality when using different filters, probably due to potential increasing solvent–filter interactions). After filling, the cartridge was degassed in vacuum for 5 min. The printing parameters were as follows: 60 °C substrate temperature, 43 °C printing head, 3 in. H_2_O meniscus vacuum set point, 40 µm drop-to-drop spacing, and ~38 V applied voltage to the printer nozzles (the applied waveform details were adapted from ref. ^43^ and can be found in the supplementary information, Supplementary Fig. 9). The printing protocol used was as follows: (1) Print 30 consecutive passes (spit-bloat cleaning step before each layer and 0.1 s purge after) with four nozzles and waiting 60 s between passes to allow solvent evaporation. (2) Reinforcement of the edges of the pattern with 18 passes (4 nozzles, same cleaning protocol, and interlayer protocol as for step #1. (3) Repeat step #1 and 2. (4) Three passes with eight nozzles without interlayer waiting or initial cleaning to achieve a wet and homogeneous surface. The layer was annealed in vacuum and darkness at 160 °C for 1 h and cooled down slowly.

*Ag contact pads*: Ag nanoparticles ink (Sicrys™ I50T-13 from pvnanocell) was sonicated for 5 min, filtered through a PTFE 0.45 µm pore-size filter and injected into a standard cartridge. The printer parameters were as follows: 55 °C substrate temperature, 33 °C printing head, 5 in. H_2_O meniscus vacuum set point, 50 µm drop-to-drop spacing, 1 nozzle, 1–2 passes, and ~25–30 V applied voltage to the printer nozzles (the applied waveform details can be found in the supplementary information, Supplementary Fig. 9). The layer was annealed simultaneously with the gate dielectric in vacuum and darkness at 160 °C for 1 h and cooled down slowly.

*Source and drain (S/D)*: Prior to (S/D) printing on the gate dielectric, the surface was O_2_ plasma treated for 20 s at 150 W. Then, PEDOT:PSS solution (Clevios PH 1000, purchased from Sigma-Aldrich) was diluted (1:3)_V_ in an aqueous solution of the ionic additive bis(trifluoromethane) sulfonimide lithium from Alfa Aesar. The final solid concentration of additive compared to PEDOT:PSS was 56 wt%). Improved droplet formation stability was achieved by adding 10% in volume of dimethyl sulfoxide (Sigma-Aldrich) to increase boiling point, and by adding 1.5 mg ml^−1^ of the surfactant sodium dodecylbenzene sulfonate to reduce surface tension. The ink was filtered through a glass fiber 0.45 µm pore-size filter and injected into a standard cartridge. The printer parameters were as follows: 40 °C substrate temperature, 32 °C printing head, 4 in. H_2_O meniscus vacuum set point, 20 µm drop-to-drop spacing, 1 nozzle, 7 passes for S/D, and 12 for the S/D edge pads, and ~25 V applied voltage to the printer nozzles (the applied waveform details can be found in the supplementary information, Supplementary Fig. 9). The ink was stable for a window of 2–3 h after filtration. After this time, nozzle clogging occurred likely due to aggregation. The channel width was 1 mm and the length was 50–100 µm.

*Channel*: SC-SWCNTs (Raymor Industries Inc. RN-020) were sorted from the metallic ones using conjugated polymer as in ref. ^44^, and flocculated as in ref. ^34^. The resulting solution with a total solid concentration of ~0.03 mg ml^−1^ of SC-SWCNTs and sorting polymer (that prevents CNTs aggregation) in toluene was mixed with a small fraction of tetralin (Sigma-Aldrich) (97.5:2.5)_V_ and the ink was sonicated for some minutes. The printer parameters were as follows: 28 °C substrate temperature, 32 °C printing head, 5 in. H_2_O meniscus vacuum set point, 20 µm drop-to-drop spacing, 2 nozzles, 8 passes, and ~25 V applied voltage to the printer nozzles (the applied waveform details can be found in the supplementary information, Supplementary Fig. 9). The ink was stable for printing during a window of 3 h after filtration. Stored in the fridge, it was usable for 1 week after sonication. After this time, nozzle clogging occurred likely due to aggregation. After channel deposition, the devices were annealed in vacuum and darkness for 30 min at 90 °C and cooled down slowly, then they were immersed for 40 s in a bath of toluene:TFA (99:1)_V_ and immediately into a second bath of fresh toluene at 100 °C and dried on a hot plate at 100 °C. Finally, the devices were annealed again in vacuum and darkness for 30 min at 90 °C and cooled down slowly to evaporate residual toluene and TFA and de-dope the SWCNTs.

*Elastomer encapsulation*: A solution of SEBS (H1062Z from Kraton™ G Polymers) was prepared in toluene (80 mg ml^−1^, stirred at 50 °C for 30 min), degassed for 5 min, and filtered through a 0.2 µm pore-size PTFE filter before spin coating it at ambient conditions at 1000 rpm for 60 s (acceleration 500 rpm s^−1^).

*Vias opening*: Toluene was filtered through a PTFE 0.2 µm pore-size filter and used to fill a standard printer cartridge. The printer parameters were as follows: 30 °C substrate temperature, 28 °C printing head, 4.5 in. H_2_O meniscus vacuum set point, 20 µm drop-to-drop spacing, 2 nozzles, 6–8 passes, and ~30 V applied voltage to the printer nozzles (the applied waveform details can be found in the supplementary information, Supplementary Fig. 9).

*Vias filling (adapted from ref.* ^38^*)*: For the formulation of solution A, multiwall unsorted carbon nanotubes (MWCNTs) (Sun Innovations SN7689): PAN (*M*_w_ = 150 kDa, Aldrich, CAS: 25014-41-9) (3:1)_wt_ were dispersed in *N*,*N*-DMF (Aldrich) (3 mg ml^−1^). Likewise, another solution (B) of graphite (Kuraray Chemical Co., Ltd. *YP-50F*):PAN (3:1)_wt_ were dispersed in DMF (3 mg ml^−1^) to obtain a graphene:PAN dispersion. Both solutions were sonicated using a Cole Parmer Ultrasonicator 750 W at 30% of maximum power for 30 min. The graphite ink was vacuum filtered through a 8 µm pore-size Teflon filter (the filter needed to be changed every few milliliters of filtered solution) and mixed (A:B) (90:10)_v_. The resulting ink was sonicated (Branson 2510 ultrasonic bath) and injected into a standard printer cartridge. The printer parameters were as follows: 50 °C substrate temperature, 33 °C printing head, 4.5 in. H_2_O meniscus vacuum set point, 10 µm drop-to-drop spacing, 4 nozzles, 65 passes with interlayer waiting time of 30 s, and ~30 V applied voltage to the printer nozzles (the applied waveform details can be found in the supplementary information, Supplementary Fig. 9). PEDOT:PSS can be also used to fill the vias using 4 nozzles and 15 passes, a drop-to-drop spacing of 10 µm, and 35 °C substrate temperature.

*Bridges and interconnects*: The same ink and parameters used for S/D were employed, except for: 35 °C substrate temperature, 30 µm drop-to-drop spacing, 3 nozzle, and 15 passes. Prior to printing, the surface was exposed to 5 s of O_2_ plasma at 60 W (higher power was shown to be detrimental for the SC-SWCNTs).

*Substrate lamination and transfer process*: A film of SEBS was fabricated by casting from solution (150 mg ml^−1^ SEBS/Toluene) on a microscope slide and letting drying at ambient conditions for 24 h; or Tegaderm^TM^ (3M) was laminated on top of the devices. Air bubbles were expelled by gently pushing with a roll. The system was heated up at 50 °C to improve adhesion and cooled down slowly before immersion in deionized water for 5–10 min until the rigid Si holder detached. The system was dried at 70 °C in vacuum and darkness for 30 min and cooled down slowly.

*Gate*: The same ink and parameters used for S/D were employed, except for: 4 nozzles and 20 passes. Prior to printing, the surface was exposed to 5 s of O_2_ plasma at 50 W (higher power was shown to be detrimental for the SC-SWCNTs).

### Device FET characterization

*FET electrical characterization*: It was performed using a Keithley 4200-SCS semiconductor parameter analyzer in ambient conditions using a gate voltage sweeping rate of 0.1 V s^−1^. The mobility has been calculated in the saturation regime as the derivative of the curve (−*I*_ds_)^1/2^ vs. *V*_gs_ according to Eq. (1). The value for each device is calculated as the average of this derivative curve over the whole range of *V*_gs_ (see Supplementary Fig. 4a), beyond the threshold voltage *V*_th_. To extract the threshold voltage, *V*_th_, we successively applied a rolling linear fitting to each 5-point segment of the curve (*−I*_ds_)^1/2^ vs. *V*_gs_. Then, *V*_th_ was assumed to be the *V*_gs_ point where the fitting line with the maximum slope cut the *x* axis. For the forward and backward voltage sweep, the threshold voltage, *V*_th_, was −0.223 ± 0.023 and 0.332 ± 0.023 V, respectively. As depicted in Fig. 3a, (*−I*_ds_)^1/2^ vs. *V*_gs_ is roughly linear only for the forward voltage sweep, which makes this value the most reliable one. The final value of saturation-regime mobility and *V*_th_ reported in the manuscript correspond to the mean value extracted from 13 single devices with the standard error. Likewise, the mobility has been calculated in the linear regime according to Eq. (2) as the mean of the derivative of the curve −*I*_ds_ vs. *V*_ds_ for *V*_ds_ > 0 (see Supplementary Fig. 4b), using *V*_gs_ = −1 V and a threshold voltage *V*_th_ = −0.2 V, which corresponds to the value extracted for forward sweeping in the saturation regime. The final value of linear regime mobility reported in the manuscript is the mean value of the averaged mobilities extracted from nine single devices with the standard error. The characterization of the FTEs under strain has been done using a stretcher instrument consisting of two clamps where the extremes of the sample were held and a micrometer screw to control the distance between the two clamps while the samples are being stretched.

The transconductance was calculated as the maximum of the derivative *−I*_ds_ vs. *V*_gs_ (Eq. (3)) for each forward and backward sweeping. The final reported value is the average from 13 devices with the standard error.

### Synaptic behavior of the FETs

It has been tested using a Keithley 4200-SCS semiconductor parameter analyzer and a pulse measure unit. Prior to the gate voltage stimulation, the device was biased at *V*_gs_ = −0.6 V, well beyond the threshold voltage *V*_th_, to ensure the complete formation of the channel and a high level of transconductance. The signals shown in the main text are the result of letting the signal stabilize on time for more than 40 s (see transient data in Supplementary Fig. 10), and filtering out the 60 Hz electrical noise using the function *sgolayfilt* of MATLAB.

### FET channel capacitance value in quasi-DC mode

It has been calculated from the charge–discharge equation of a parallel-plates capacitor as explained in ref. ^31^. A constant voltage was applied with a Keithley 2400 source meter across the capacitor connected in series with a known resistor, while the voltage increase across the capacitor was measured using a Keithley 2636A source meter.

### Mechanical characterization

The stress–strain curves were obtained using an Instron pull tester at a strain rate of 10% strain min^−1^.

## Supplementary information

Supplementary Information

## Data Availability

The authors declare that most data supporting the findings of this study are available within the paper and its supplementary information files. The rest of the data are available from the corresponding author upon reasonable request.
