# Supplementary material for: Inkjet-printed stretchable and low voltage synaptic transistor array
Source: Nat Commun. 2019 Jun 18;10:2676. doi: 10.1038/s41467-019-10569-3 (PMC6582140; doi:10.1038/s41467-019-10569-3)
Supplement: Supplementary file 1 — Supplementary Information [file 41467_2019_10569_MOESM1_ESM.docx]

**Supplementary Information**

**Inkjet-printed stretchable and low voltage synaptic transistor array**

Molina-Lopez et al.

**Supplementary Figures**


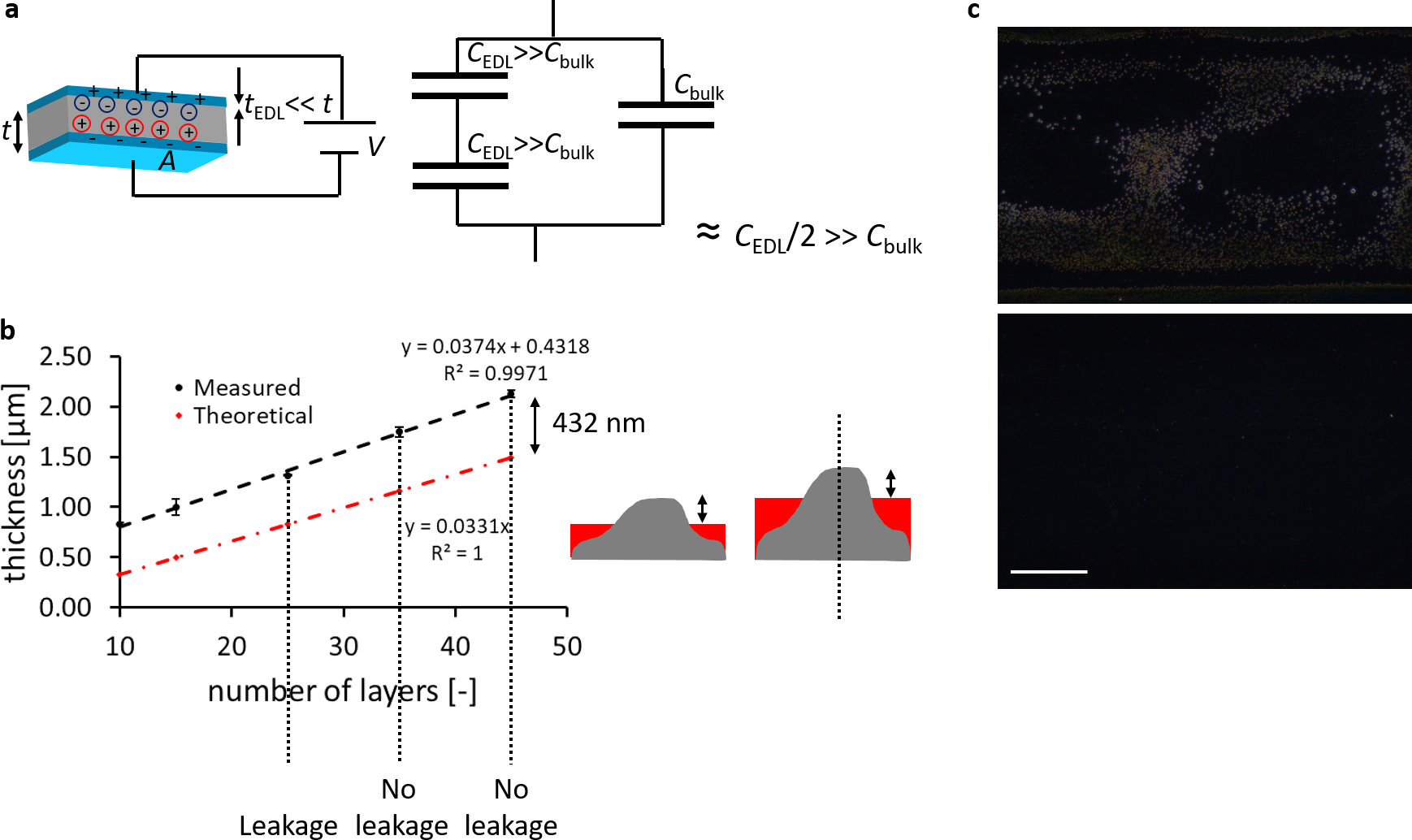


**Supplementary Figure 1. Inkjet-printed PVDF-HFP for capacitors with EDL effect. a)** Sketch representing the electrical double layer (EDL) effect in a parallel plate capacitor and electrical model of the EDL capacitor: ions migrate to the interface with the oppositely charged electrode creating an ionic layer that acts as a virtual electrode. Large capacitance *C*_EDL_ appears as a consequence of the short separation (molecular distance) between charges *t*_EDL_. Hence the bulk dielectric thickness *t* becomes irrelevant in the final capacitance value. (Note that the normal bulk dielectric-related capacitance of a parallel plate capacitor is *C*_bulk_ *= ɛɛ_0_ A/t* with *ɛ* and *ɛ_0_* being the permittivity of the dielectric and vacuum, respectively; *A* the surface area of the electrodes and *t* the distance between electrodes). The temperature dependency of PVDF-HFP has been reported in the supplementary info of reference ^1^, by measuring the capacitance of a capacitors using PVDF-HFP as dielectric layer. The capacitance, both in AC (measured with a LCR-meter at 735Hz) and quasi-static (measured using RC charging) mode, was unchanged from ~ -20°C to 55°C. Below -20°C, the capacitance showed a sudden drop corresponding to a glass transition that reduced the movement of the ions and the formation of the (EDL). **b)** Thickness versus number of stacked printed layers for the ionic PVDF-HFP gate dielectric and minimum thickness leading to no gate current leakage (1.75 µm). The shift between the theoretical thickness (black dashed line, calculated as in ^2^) and the measured thickness (red dashed line, error bar is standard error) is a result of the non-uniform thickness of the film as represented by the sketch on the right. In the sketch, the theoretical film is depicted in red and the actual film in gray. The dash line represents the point where the actual thickness was measured. However, both curves show similar slope, indicating a thickness increase of 37.4 nm per printed layer. **c)** Dark field microscopy pictures of representative rough (top) and smooth (bottom) inkjet-printed PVDF-HFP surface. PEDOT:PSS lines with straight edges could not be printed on the rough surface on the top side. Scale bar = 200 μm.


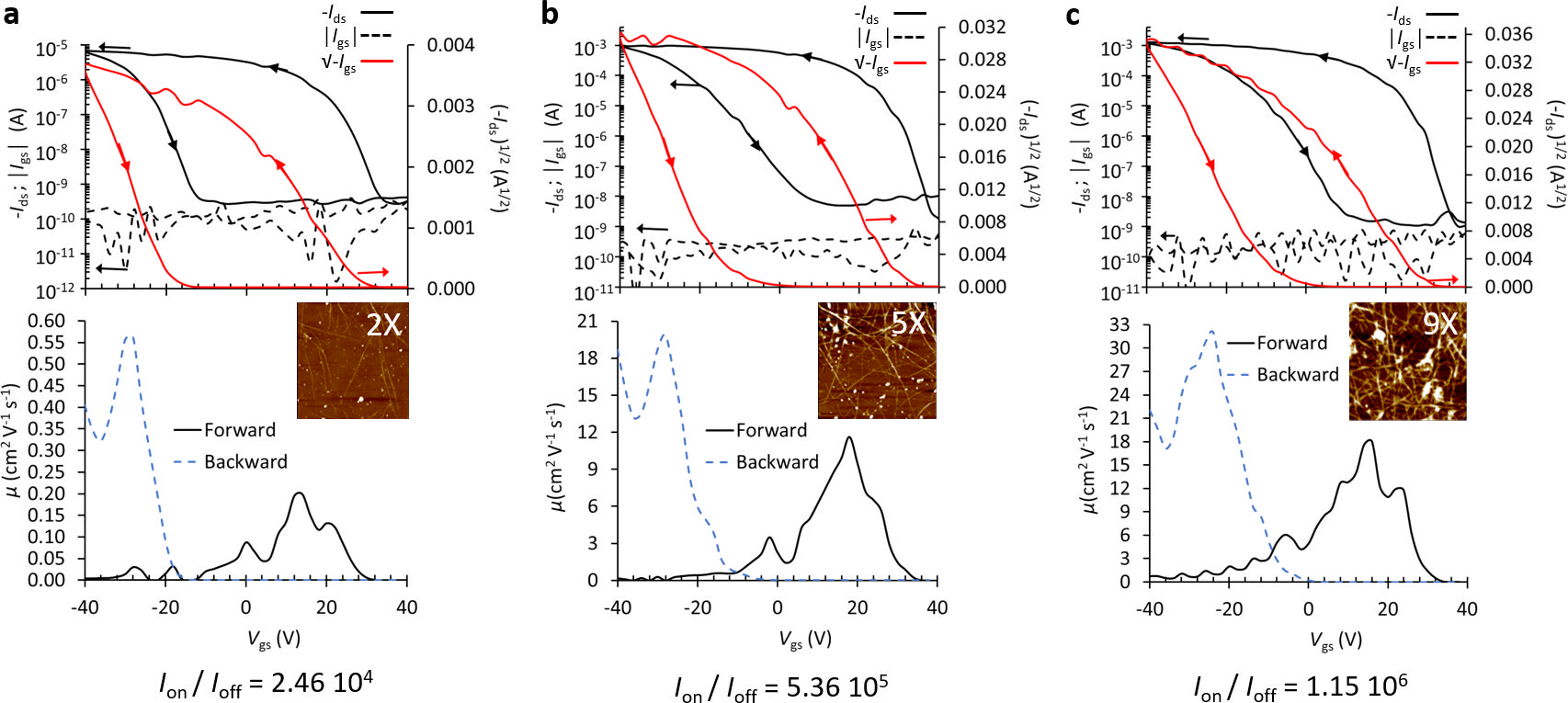


**Supplementary Figure 2. Electrical performance of printed SC-SWCNTs vs printing passes.** Electrical characterization of sorted SC-SWCNTs FETs inkjet-printed on SiOx (300 nm - thick) for 2 **(a)**, 5 **(b)** and 9 **(c)** printing passes, respectively. Mobility has been calculated with the parallel plate capacitance model for simplicity using a channel width and length of W = 2.1 mm and L = 120 μm, respectively. Top contact source and drain electrodes were fabricated from IJ-printed Ag annealed in vacuum at 160°C. The sorting polymer was removed by immersion in toluene:trifluoroacetic acid (TFA) (99:1)_V_ after annealing.


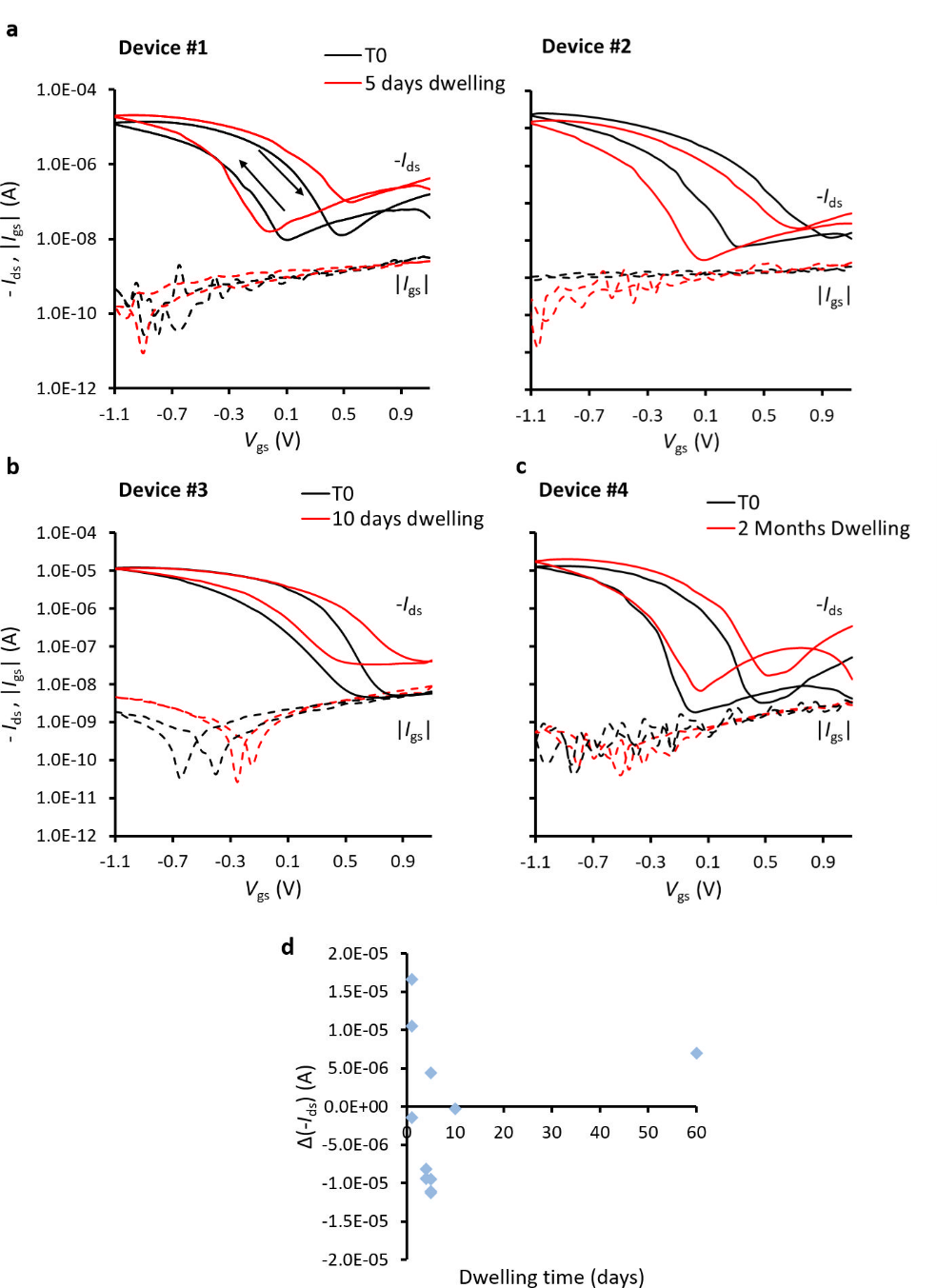


**Supplementary Figure 3. Ambient stability of the printed SC-SNWTs on PVDF-HFP.** Transfer curve of representative devices measured immediately after fabrication and after dwelling in either ambient conditions for 5 days (Device #1 and #2 ) **(a)**; or partly ambient / partly vacuum conditions for 10 days (Device #3) **(b),** and 2 months (Device #4) **(c)**. No significant degradation of the on/off current ratio, mobility or gate leakage current was observed after dwelling. The slight shifts in threshold voltage and maximum on-current observed before and after dwelling do not show any trend. Hence, they are likely due to the uncontrolled memory effect of the ionic gate dielectric and the measurement noise introduced by un-optimized electrical contacts and interconnections. **d)** Difference in maximum on-current, *Δ(–I*_ds_*)*, after several dwelling times for 12 devices. A positive (negative) *Δ(–I*_ds_*)* indicates that the current is higher (lower) after dwelling.


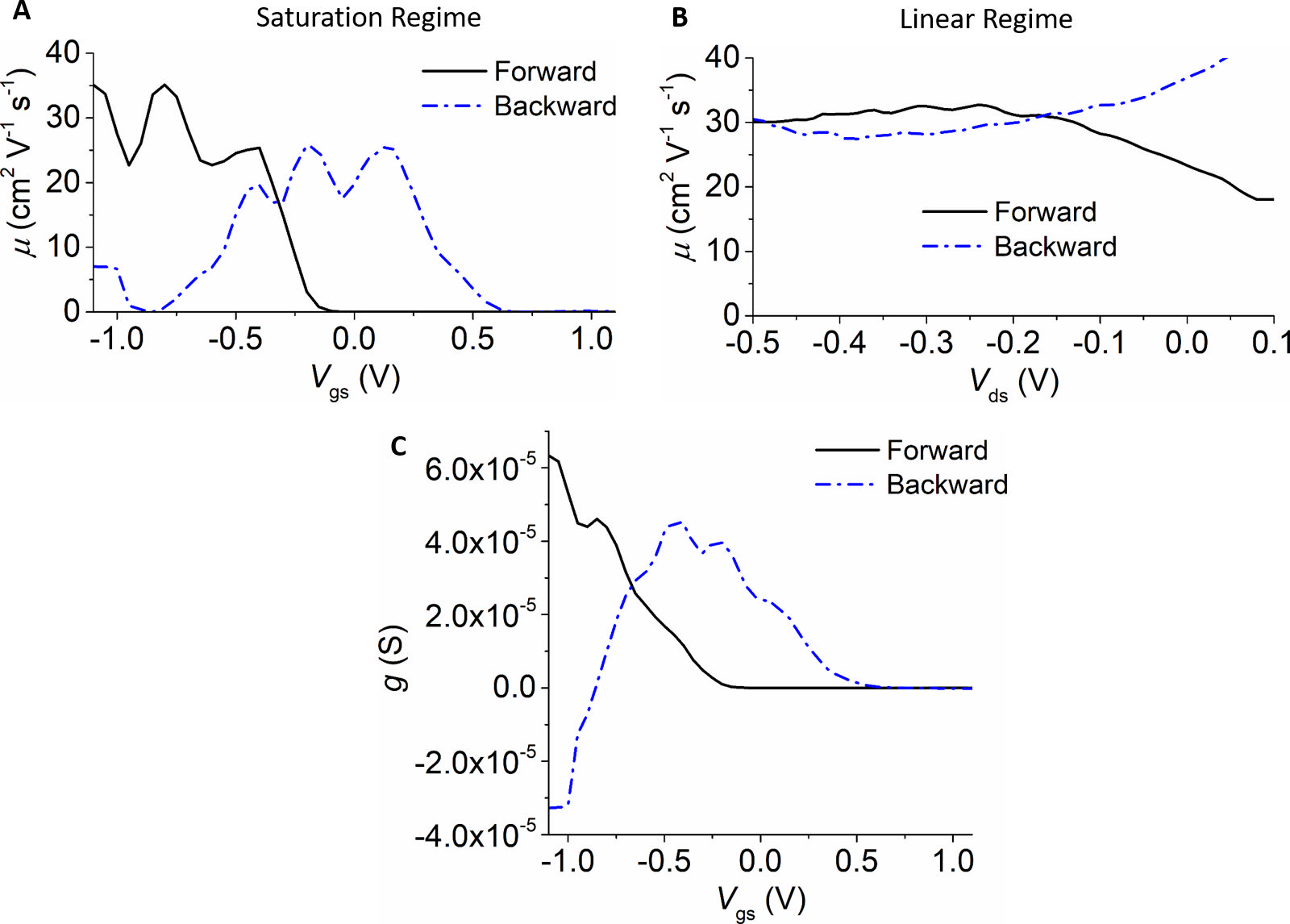


**Supplementary Figure 4. Electrical performance at different gate voltages. (a)** Values of mobility in saturation regime versus gate voltage (forward and backward sweeping at *V*_ds_ = -1.1 V), and **(b)** in linear regime versus drain-source voltage (at *V*_gs_ = -1 V), for the same representative inkjet-printed stretchable ionic transistor. **c)** Values of transconductance versus gate voltage (forward and backward sweeping at *V*_ds_ = -1.1 V) for the same device.


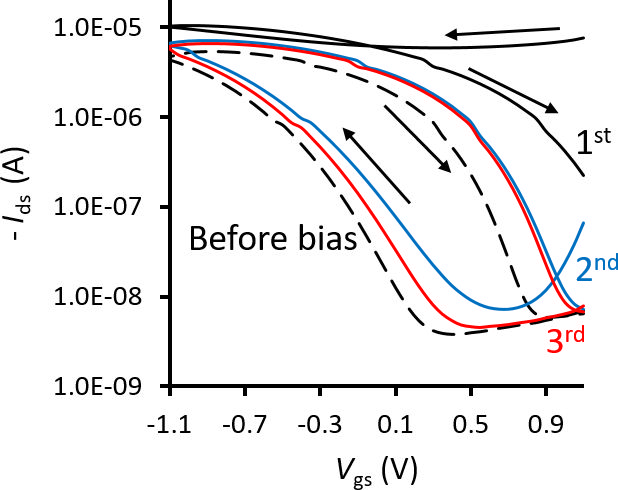


**Supplementary Figure 5. Memory effect of the transistors.** Transfer curve of a representative working device before (dash line) and after holding the gate voltage at -20V for 2 seconds. The transistor behavior is temporarily lost right after strong biasing the gate (1st measurement). However, the transistor behavior is recovered after immediately re-measuring the device (2nd and a 3rd time) and the threshold voltage gets progressively closer to its original value after each measurement.


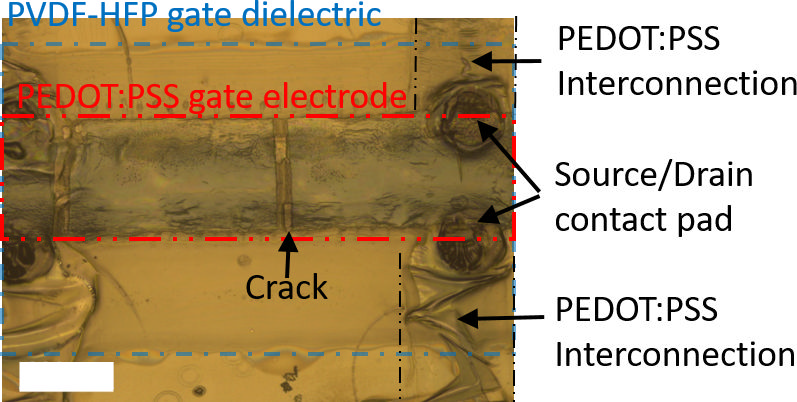


**Supplementary Figure 6. Gate crack failure mode.** Optical picture showing gate electrode crack beyond 20% strain. The surface where the gate electrode was printed corresponds to the side of the gate dielectric that was in contact with the sacrificial layer before releasing the system from its rigid carrier. Therefore, this surface is different from the surface where the S/D were printed. It was harder to get uniform films with straight edges for the gate electrode than for the S/D electrodes. We believe that such imperfections promoted stress concentration and resulted in crack initiation and propagation at lower strain for the gate electrode than for the S/D electrodes (Supplementary Figure 8). Scale bar = 200 μm.


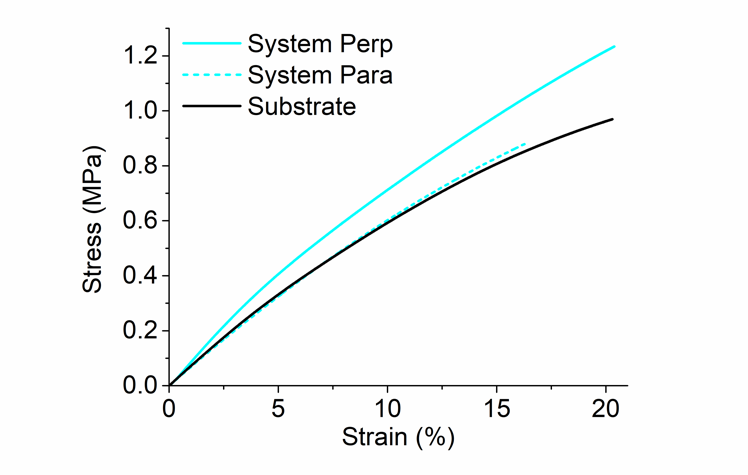


**Supplementary Figure 7. Tensile Stress-Strain curves of the fabricated system and comparison with the SEBS substrate.** The Young modulus *E* for the system along the direction parallel to the channel length was identical to the substrate modulus: *E_para_* = *E_subs_* = 6.3 MPa. The Young modulus along the direction perpendicular to the channel length was slightly higher, likely due to the PEDOT:PSS electrodes: *E_subs_* = 7.6 MPa. The modulus has been extracted from the slope of the curves up to 10% strain.


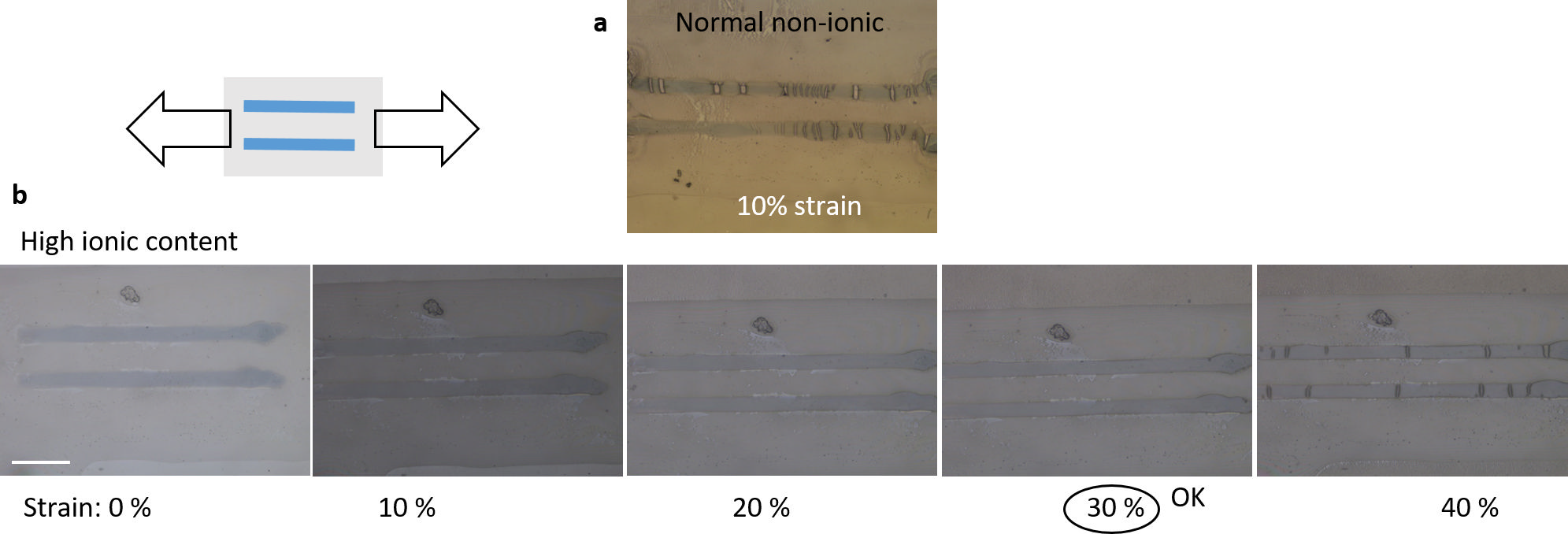


**Supplementary Figure 8. Stretchability of ionic PEDOT:PSS.** Difference in stretchability between source and drain electrodes printed with non-ionic **(a)**, and highly-ionic PEDOT:PSS **(b)**. The highly-ionic material can be stretched to 30% of its original length before cracking*.* Scale bar = 200 μm.


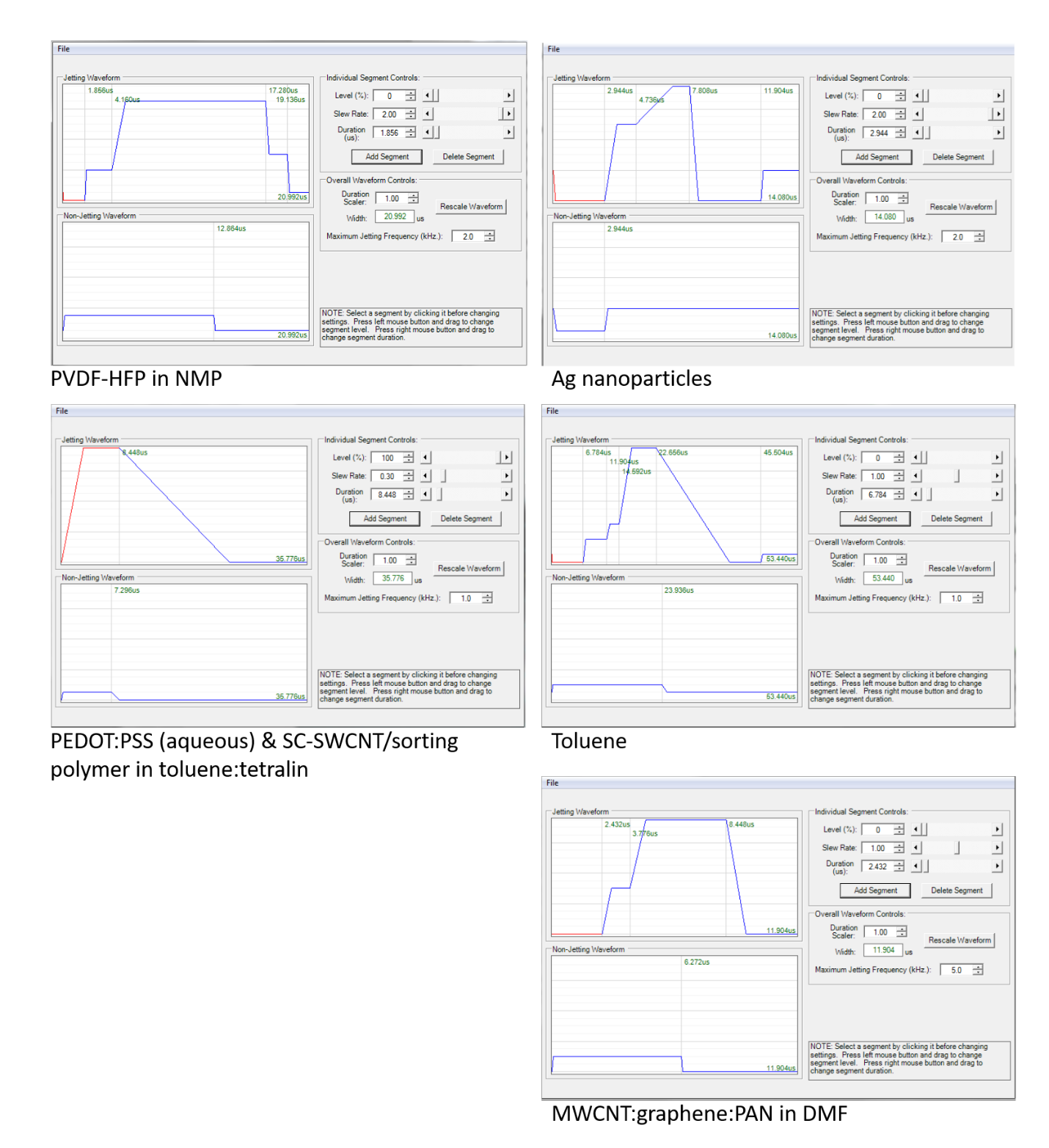


**Supplementary Figure 9. Jetting waveform settings used to inkjet-print each material.** Waveforms applied to the printer (*Fujifilm Dimatix* DMP-2831) nozzles for every inkjet-printed material.





**Supplementary Figure 10. Synaptic behavior of the printed FETs (transient raw data).** Transient raw data corresponding to the source-drain current (*I*_ds_, on the left axis) response over time to small gate voltage pulses (*V*_gs_, on the right axis) that imitates neuron pre-synaptic potential spikes for the IJ-printed intrinsically-stretchable devices (W / L = 1000 µm / 50 µm). The pulses consist of a square signal of -80 mV of amplitude, 25 Hz of frequency and duty cycle of 50%. The drain-source voltage was held at -1.1 V. The source-drain current response is tested for consecutive trains of 1, 1, 2, 2, 2, 16 and 32 gate voltage pulses, displaying typical postsynaptic current that increases with the number of pulses and slowly relaxes in their absence. The FET was biased at a dc gate voltage of -0.6 V to ensure the formation of the channel. However, the slow movement of the gate dielectric ions leads to a long stabilization times as reflected by the drift of the baseline of the source-drain current. After ~ 40 seconds, the baseline of the signal begins to stabilize and the device is ready to operate as a synaptic FET. The superposition of the short-term response of the channel to the small gate pulses and the longer-term response to the bias voltage (the baseline current that tends to stabilize) can be observed before 40 seconds. This superposition suggests that the devices are capable of mimicking both short-term and long-term plasticity, depending on the amplitude of the voltage applied to the gate.

**Supplementary References**

1. Kong, D. *et al.* Capacitance Characterization of Elastomeric Dielectrics for Applications in Intrinsically Stretchable Thin Film Transistors. *Adv. Funct. Mater.* **26**, 4680–4686 (2016).

2. Salmerón, J. F. F. *et al.* Properties and Printability of Inkjet and Screen-Printed Silver Patterns for RFID Antennas. *J. Electron. Mater.* **43**, 604–617 (2014).
